# Advanced manufacturing provides tailor-made solutions for crystallography with x-ray free-electron lasers

**DOI:** 10.1063/4.0000229

**Published:** 2024-02-21

**Authors:** Lars Paulson, Sankar Raju Narayanasamy, Megan L. Shelby, Matthias Frank, Martin Trebbin

**Affiliations:** 1 Department of Chemistry & Research and Education in Energy, Environment and Water (RENEW), The State University of New York at Buffalo, Buffalo, New York 14260, USA; 2 Biosciences and Biotechnology Division, Physical and Life Sciences Directorate, Lawrence Livermore National Laboratory, Livermore, California 94550, USA; 3 Department of Biochemistry and Molecular Medicine, School of Medicine, University of California, Davis, California 95817, USA; 4 Hauptman-Woodward Medical Research Institute, Buffalo, New York 14203, USA

## Abstract

Serial crystallography at large facilities, such as x-ray free-electron lasers and synchrotrons, evolved as a powerful method for the high-resolution structural investigation of proteins that are critical for human health, thus advancing drug discovery and novel therapies. However, a critical barrier to successful serial crystallography experiments lies in the efficient handling of the protein microcrystals and solutions at microscales. Microfluidics are the obvious approach for any high-throughput, nano-to-microliter sample handling, that also requires design flexibility and rapid prototyping to deal with the variable shapes, sizes, and density of crystals. Here, we discuss recent advances in polymer 3D printing for microfluidics-based serial crystallography research and present a demonstration of emerging, large-scale, nano-3D printing approaches leading into the future of 3D sample environment and delivery device fabrication from liquid jet gas-dynamic virtual nozzles devices to fixed-target sample environment technology.

## INTRODUCTION

Biophysical techniques, such as macromolecular x-ray crystallography (MX)^1,2^ and cryogenic electron microscopy (cryo-EM), have been actively utilized to investigate the structural mechanisms of viral pathogenesis and develop therapeutics and vaccines. Knowledge of the 3D structures of disease-relevant proteins, their dynamic processes, and the connection between structure and function can greatly accelerate the rational design of novel and improved drug candidates and provide insight into their roles in pathogenesis. In contrast to approaches that use binding affinity alone to assay drug or therapeutic effectiveness, crystallography of protein-ligand complexes determines the 3D conformation of the protein binding site and provides complementary information relevant for structure-based and fragment-based drug design.

Although conventional, cryogenic x-ray crystallography and cryo-EM techniques currently account for the vast majority of structures solved, there are many samples that are less tolerant to radiation damage, such as certain metalloenzymes,^3^ making measurements from a single stationary sample intractable despite damage-mitigating cryogenic temperatures. Room-temperature protein crystallography provides a means to elucidate protein structure and function under more physiologically relevant (e.g., not cryogenic) conditions. Specific applications also preclude cryogenic temperature measurements. In particular, investigations of protein dynamics and time-resolved studies on short timescales require the sample to be at room temperature in solution phase, rather than frozen, thus tremendously profiting from measurements at room temperature.^4–8^ Dynamic, time-resolved room-temperature crystallography^8–12^ can provide additional information such as mechanistic studies of protein inhibition processes related to the protein–ligand complex formation and, thus, aids drug discovery.^13,14^ These technical limitations can be addressed using serial femtosecond crystallography.^15^

Serial femtosecond crystallography (SFX) using an x-ray free-electron laser (XFEL) provides an alternate macromolecular crystallography approach in which a series of individual diffraction frames are measured from a constant stream of microcrystals in random orientations. SFX enables the determination of room-temperature structures without the effects of radiation damage influencing the measured diffraction (i.e., “diffraction-before-destruction”).^16^ The intensity and focus of the XFEL beam also enables the determination of high-resolution room-temperature structures for difficult-to-crystallize proteins, including many membrane proteins, using microcrystals. Moreover, using optical pump-probe or mix-and-injection approach, SFX enables the measurement of conformational changes induced by the light or mixing trigger over a wide time range and facilitates determination of intermediate state structures during a molecular reaction. Some ligand-binding or virulence-relevant processes may also be less conventionally triggered with light,^17^ e.g., through the use of caged (ligand) compounds that can be diffused into crystals in caged (inactive) forms and uncaged (activated) by a light pulse.^18^ In case of serial synchrotron crystallography (SSX), the x-ray dose is spread over multiple crystals rather than a single one, benefiting the collection of low-dose room-temperature data.^19^ In serial crystallography methodologies, small dataset is collected from each crystal, and the collection of partial data is evaluated and merged together to generate complete dataset. Hence, for these experiments that involve time-resolved mixing component, well-controlled *in situ* crystallization, and for other biochemical reactions, large quantities of small crystals needs to be handled with micrometer precision, and their corresponding solutions (buffer solution).^20^

Based on the x-ray beam properties, the typical crystal size range can vary for serial crystallography experiments at XFELs and synchrotrons from 1 to 150 *μ*m.^21–23^ To minimize both sample consumption and contributions to background due to the presence of excess solvent in the beam, sample delivery should ideally involve precise handling of nano-to-microliter volumes. Serial crystallography requires high-throughput sample introduction of small crystals in small volumes into the x-ray beam. This can be realized and optimized for low sample consumption through microfluidic methodologies. Microfluidic-based sample environment and delivery strategies have the option to include advanced sample handling on-the-chip (e.g., mixing, switching, sorting, trapping), thus providing valuable tools for data collection at brilliant x-ray light sources.^24,25^ However, traditional fabrication methodologies for microfluidics, such as standard soft lithography, allow for only 2D limited device features and functionality, while still requiring manual steps and long manufacturing times. To address this challenge, researchers from the NSF BioXFEL Center^26^ have started utilizing advanced 3D fabrication technologies, such as two-photon polymerization (2PP), projection micro-stereolithography (P*μ*SL), and laser ablation, to achieve feature definition down to sub-micrometer resolution.^27–31^ Initially, these methodologies were only implemented to produce small nozzles for liquid jet-based sample delivery methods, but they are now being applied for fabrication of a much wider range of microfluidic devices such as in fixed-target sample delivery platforms.

Liquid jets, though a proven delivery method for both static and time-resolved experiments,^9,32,33^ have some limitations in sample-limited situations. Over the period of several experiments,^34–38^ the authors have demonstrated fixed-target delivery strategies with lower sample consumption rates, higher hit rates, and simplified sample handling. However, the spatial resolution of complex microfluidic sample handling technologies has, so far, not approached the micrometer range to match typical crystal dimensions. The challenge lies within non-linear scaling of 3D printing times with increasing volume, especially for 2PP. In addition to high spatial resolution, a fixed-target working area must typically span several millimeters, or even centimeters, because it is required to maximize data collection from a single fixed-target chip before having to exchange devices. Thus, a main limitation has been the relatively small maximum printing area and slow printing speed of two-photon polymerization by nano-3D printers. Here, we discuss advances in the field of serial crystallography at XFELs and synchrotrons in liquid jet sample delivery using high-resolution 3D printing and present a novel sub-micron-resolved 3D printing technology using rapid two-photon polymerization methodology to print over a 16 × 16 × 3.5 cm^3^ volume in a reasonably short time (i.e., seconds to minutes) that can be utilized for fixed-target platforms. This new advanced manufacturing capability will facilitate fabrication of future complex liquid jet devices and 3D printing based fixed-target sample environments, enabling high-throughput structure determination experiments at XFELs and synchrotrons.

## OVERVIEW OF SAMPLE ENVIRONMENTS FOR SERIAL CRYSTALLOGRAPHY

**FIG. 1.**
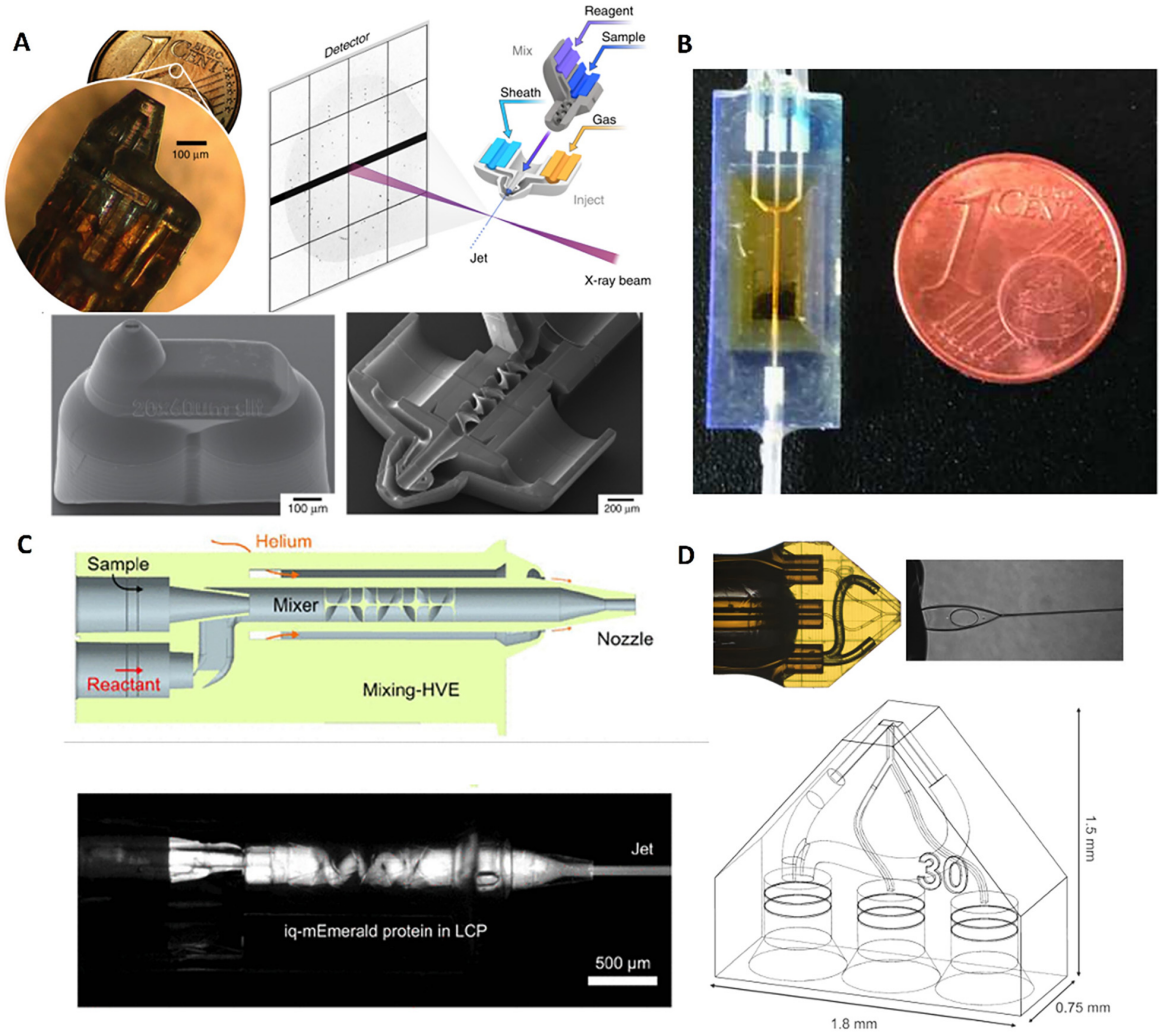
Overview of various 3D printing efforts for serial crystallography at XFELs and synchrotrons. (a) Two-photon polymerization (2PP)-based gas-dynamic virtual nozzles (GDVN) with mixing elements [Reproduced with permission from J. Knoška, *et al.*, Nat. Commun. **11**, 657 (2020). Copyright 2020 Authors, licensed under a Creative Commons Attribution (CC BY) license];^28^ (b) Projection microlithography-based hydrodynamic flow focusing chip for serial crystallography experiments [Reproduced with permission from D. C. F. Monteiro *et al.*, IUCrJ **7**, 207 (2020); Copyright 2020 Authors, licensed under a Creative Commons Attribution (CC BY) license];^24^ (c) 2PP fabrication-based extrusion nozzle for Lipidic Cubic Phase (LCP) samples [Reproduced with permission from M. Vakili *et al.*, J. Appl. Crystallogr. **56**, 1038 (2023). Copyright 2023 Authors, licensed under a Creative Commons Attribution (CC BY) license];^29^ (d) 2PP fabrication-based flat jet nozzle [Reproduced with permission from P. E. Konold *et al.*, IUCrJ **10**, 662 (2023). Copyright 2023 Authors, licensed under a Creative Commons Attribution (CC BY) license].^49^ 2PP fabrication has significantly advanced the sample environment and delivery for serial crystallography experiments.

Serial crystallography experiments require continuous sample replenishment, which plays a crucial role in the avoidance of radiation damage impacts on the data. Common strategies for SFX sample delivery can be broadly classified into liquid jets,^27,28,31,39–41^ fixed targets,^34^ and tape-drives.^42^ The liquid jets can consist of monodisperse droplets,^27^ homogeneous droplets,^43^ with population of discrete sizes, gas focused spray, electrospray,^44^ flat jets,^32^ or lipid cubic phase injectors.^45^ Liquid jets can include mixing elements^28^ to perform time-resolved studies.^46^ Fixed targets can be open-to-air^34^ or sealed within polymer^47^ or other composite films.^48^ For fixed-target sample environments, there is also the added requirement of maintaining hydration of the biological samples during sample storage and measurement. This requirement can be accomplished, for example, by sandwiching thin, well-defined sample layers in between ultrathin polymer windows with high barrier properties as previously demonstrated at XFELs and synchrotrons.^37,38^ Beyond serial crystallography, ultrathin low-background sample environments, such as FT devices and liquid sheet jets, also offer exciting opportunities for (soft) x-ray spectroscopies (e.g., at ChemRIXS) and fluctuation and conventional solution scattering.^38,49,50^ Owing to the continuously varying demands of the sample types, mixing times, and other challenges such as clogging and excessive sample consumption, 3D printed solutions have increasingly been integrated as components of the XFEL sample environment, either in the form of nozzles, mixing elements,^28^ catchers, aerodynamics lenses,^51^ or fixed-target support structures (Fig. 1).

While the first XFELs have operated at moderate pulse repetition rates of ∼30–120 Hz, newer XFELs operate at much higher rates of kHz up to MHz,^52–54^ which require much faster sample replenishment. To meet this challenge, solutions using facile sample environment development and high-throughput delivery have become more important than ever. Adaptable fabrication methods for sample delivery devices also facilitate tailoring to unique experimental and facility needs. Here, we will dive into the current needs and future recommendations for the development of advanced fixed-target sample environment solutions and how advanced manufacturing technologies such as 3D printing can bridge the gaps.

## EVOLVING MANUFACTURING TECHNOLOGIES

The first widely used sample injectors for XFELs were gas-dynamic virtual nozzles made from glass capillaries.^39^ These devices had two major advantages: (1) The gas focusing results in a smaller liquid stream than the outlet diameter; and (2) glass capillary tubing is widely available and the manufacturing is done with in-laboratory methods. Because these devices were handmade and required careful handling and custom polishing, the manufacturing was non-homogeneous and slow.^39,55^ Furthermore, more complex devices, such as capillary-based mix-and-inject gas-dynamic virtual nozzles (GDVNs), can have more than 100 fabrication steps^33,41^ increasing fabrication difficulty, error rate, geometric variations, and manufacturing time.

Later, polydimethylsiloxane soft-lithography GDVNs were developed pioneering the age of CAD-based sample environments and microfluidic on-chip mixing GDVNs.^40^ The first PDMS devices had a similar GDVN design as initial capillary devices, but moving from glass capillary manufacturing to microfluidic soft lithography allowed the creation of arbitrary CAD geometries, as demonstrated by the on-chip mix-and-inject design.^40^ Thus, soft lithography opened the door for future designs to be tailored to sample delivery. Traditional lithography relies on two-dimensional masks (i.e., physical or projected), which can limit the achievable resolution, constrain the freedom to design 3D structures, and make the stacking of layers on a wafer a tedious process.^40^

Now, less than two decades after GDVN capillaries were introduced, the XFEL sample environment community has access to commercial two-photon 3D printers capable of fabricating nearly arbitrary 3D structures with micrometer-scale resolution. These abilities greatly expand the possible device geometries, including helical mixers in a modular configuration,^28^ asymmetric liquid outlets,^27^ and high-viscosity sample injectors.^29^ In direct laser writing (DLW) method, the printing time of two-photon printers scales with the total volume written. Because of this, the usual practice is to minimize the printed volume of two-photon devices to reduce the writing time and allow fabrication of multiple devices within reasonable times, i.e., seconds to minutes. A single GDVN can require several hours to print using a commercial two-photon instrument.^27,29,30^ When an XFEL beamtime requires dozens of sample injectors and fixed-target platforms, reducing the device size and fabrication time as much as possible is critical to completing device production in a timely manner. Knoška *et al.*^28^ have drastically reduced writing time using a combination of: (1) varying the laser power and corresponding voxel size depending on the resolution required by the device geometry; and (2) using a shell-scaffold model, followed by UV curing, to reduce internal printing volume. The combination of these two techniques reduces writing time from hours to minutes, which is an outstanding achievement. However, the device size is still limited, i.e., the largest reported size is only several cubic micrometers, and assembly and integration difficulties remain.^30^ For example, the 3D-printed GDVN devices still have to be assembled by manually epoxy-gluing them onto capillaries. Hence, implementing the existing commercial two-photon polymerization methodology for fixed targets, which require larger print area, would not be realistic. While we emphasize miniaturization and the ultracompact nature of the designs, this motivation is driven not only by the desire to reduce printing times but also to increase device efficacy and efficiency for x-ray data collection applications.^56^

Modular connections reduce integration difficulties,^28^ but beamtime can still be lost due to the disconnection of tubing when replacing broken or clogged devices during shifts.^57^ A microfluidic solution for this issue could be the use of dense manifold connectors, internal microvalve switches, and addressable GDVN nozzle arrays.^58^ Alternatively, fixed-target sample environment platforms solve the difficulties of microfluidic connections by eliminating the extended capillary connections altogether and have seen increasing adoption for serial crystallography experiments at XFELs and synchrotrons. These devices hold large numbers of individual microcrystals, allowing individual data collection or spatial rastering.^37,38^ Originally, the fixed-target platforms were made from silicon,^59^ now their relatively simple designs are usually manufactured with laser ablation or other subtractive manufacturing techniques, using wide range of materials including Kapton, Mylar, and cyclic olefin copoylmers.^16,37,38^ This material variety can tailor devices to x-ray characteristics and sample environment needs.^47,48^

## FUTURE OF 3D PRINTING TO ACCOMMODATE THE NEEDS OF XFEL RESEARCH

Surprisingly, fixed-target sample environment platforms, despite their demonstration of high-hit rates, *in situ* crystallization, and shear sensitive sample handling, have not fully utilized the range of fabrication opportunities offered by 3D printing. The implementation of 2PP manufacturing for fixed targets would require printing over a very large area, nearly 10–20 times larger than the existing printing region of most commercially available 3D printers. Overcoming these limits will be the key to creating the next generation of microfluidics-enabled, 3D printed, fixed-target sample environment platforms and liquid jet injectors.

**FIG. 2.**
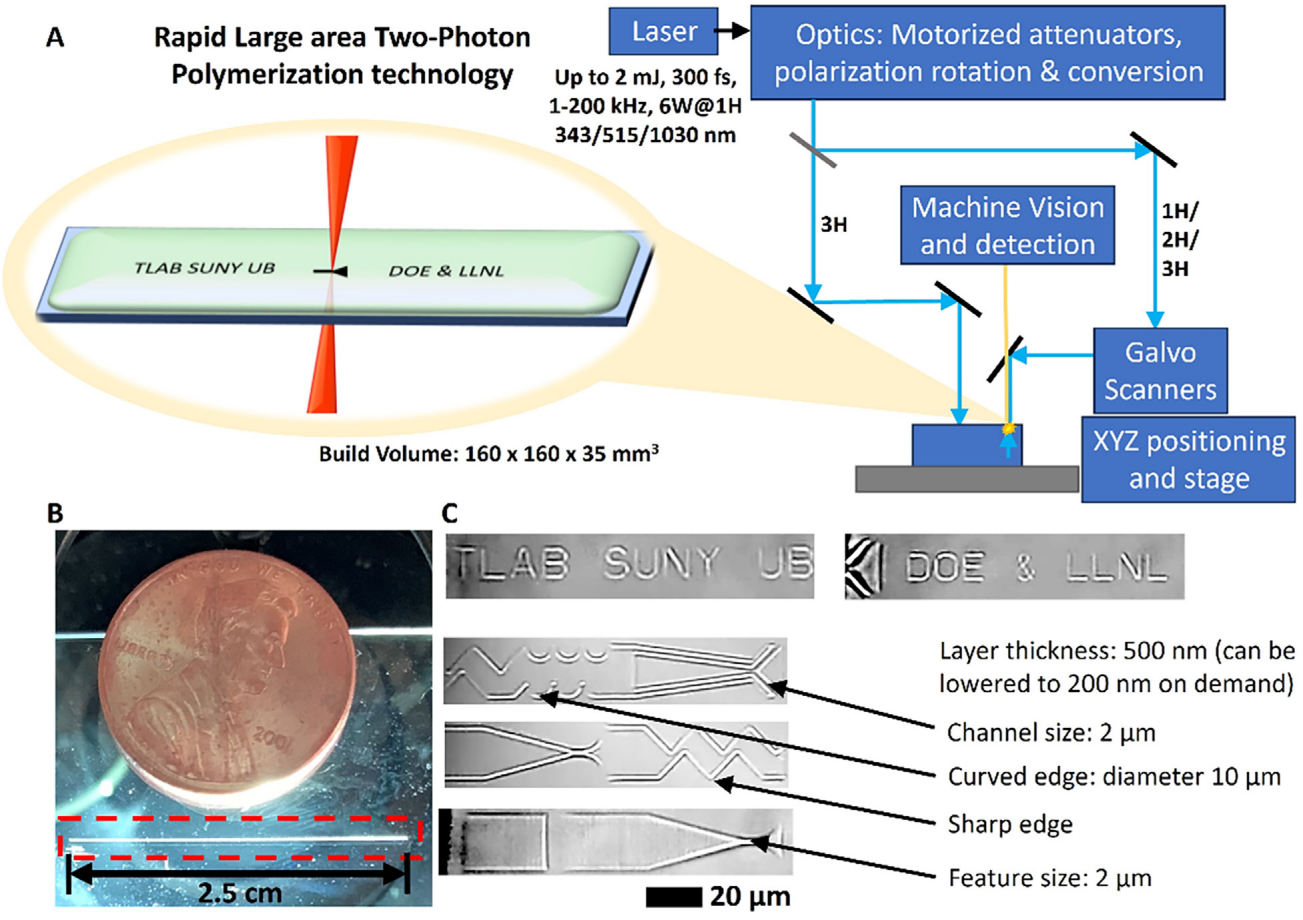
Rapid large-area two-photon polymerization: (a) Schematic representation of large-area 2PP: here the features are enlarged for artistic rendition. The femtosecond laser-based rapid large-area 2PP instrumentation schematic representation. This setup has a very large area of up to 160 × 160 mm^2^ with the ability to print intricate sub-micron features. (b) Printing demonstration of the new rapid large region 2PP setup with the features required for fixed-target sample delivery, i.e., a minimum length of 500 *μ*m and features smaller than 5 *μ*m. As a demonstration, sub-micron featured structure is printed over a very long range of 2.5 cm (compared to a penny). The layer thickness can be custom adjusted on-demand for each feature, down to the sub-micron range. In these examples, the layer thickness is greater for visibility. (c) The printing ability is shown in live printing videos: Supplementary video 1 (Ref. 71) showing one pair of structures over a length of 500 *μ*m in real time (15 fps) and supplementary video 2 (Ref. 71) showing repeated structures over a length of 2.5 cm captured at 60 fps over a period of 34 min, which is shown with 30× accelerated speed.

Improvements in the maximum build area of commercial two-photon printers, such as the latest configurations of the Nanoscribe and UpNano printers,^60,61^ as well as the in-house system shown in Fig. 2(a), have created the possibility of swiftly fabricating fixed-target geometries featuring microfluidic functional elements. By writing precise microstructures over a large (few-cm^2^ scale) working areas [Figs. 2(b) and 2(c)], comparable to the total size of fixed-target devices, these emerging large-area fabrication methods can create fixed targets optimized for advanced sample handling on-the-chip. Specifically, user friendliness and earlier stages of sample preparation can be addressed, such as enabling protein crystallization directly on the chip, thus eliminating the need for crystal transfer and enabling *in situ* measurements. Furthermore, fixed-target devices, modular features to avoid x-ray damage to neighboring crystals, for crystal trapping and microfluidic mixing, would require precise fabrication with a size range of 1–50 *μ*m.

An emerging alternative to the 2PP-3D-microfabrication of XFEL sample environments is projection microstereolithography (P*μ*SL) because it is much cheaper and more widely available than two-photon systems.^56^ Unlike 2PP instruments, P*μ*SL-3D-printers do not raster-scan a single highly focused beam, but expose the photo resin to a uniform projection of the desired features simultaneously. While P*μ*SL-3D-printers have achieved single-micron printed features,^62^ the high-resolution projected pixel pitch typically comes at the cost of a reduced exposed area due to the fixed size of the pattern-generating digital mirror device (DMD).^24,58^ While the resolution should in principle be sufficient to 3D-microprint GDVN geometries, it remains to be seen if integrated geometries with increased complexity, such as 3D micromixers, can be realized. An exciting prospect is the combination of 2PP and P*μ*SL: the bulk geometry could be 3D-printed at high volume rates using P*μ*SL, and complex features could be added using 2PP, as demonstrated in silicon,^63^ glass,^64,65^ or microstereolithography^66^ fabricated chips.^67^

However, one serious limitation of this method will remain: as a layer-based printing process, each layer must be sufficiently supported, making the possible 3D geometries more restricted than those achievable with DLW methods. For complex 3D structures such as high-performance mixers,^28^ P*μ*SL resolution advances will not suffice. Fortunately, two-photon DLW methods are also making rapid capability improvements. In addition to the drastic increase in fabrication speed discussed earlier, commercial manufacturers have improved build volume, performance and developed new capabilities such as writing objects inside an existing microfluidic channel. There has also been research into combining various printing methods to create a single device—using DLW methods only for the high-resolution sections, and a faster and cheaper alternative for the rest, for example, embedding a DLW-fabricated GDVN into a silicon,^63^ glass,^64,65^ or microstereolithography^66^ fabricated chip. If a smaller diameter outlet than can be comfortably printed as necessary, additive manufacturing techniques can fabricate most of the device, with high-resolution laser ablation for the outlet.^67^

## OUTLOOK

Over the last decade, room-temperature serial crystallography at synchrotrons and XFELs has evolved and provided exciting new capabilities for dynamic structure determination of proteins and other macromolecules. These include the ability to obtain high-resolution, near damage-free, room-temperature structures from challenging crystallization targets, including membrane proteins, to perform time-resolved structural studies on conformational changes induced by light or ligand-binding over a wide time range from sub-picoseconds to milliseconds, and, ultimately, to produce the first molecular movies of biomolecules in action. Developing new, tailored sample delivery techniques and platforms have played a crucial role in these accomplishments. Various sample environment research groups^30,37,38,68–70^ continue collaborative work addressing emerging sample introduction needs for high-repetition rate XFELs, such as the EuXFEL, LCLS-II (Linac Coherent Light Source II), and the compact CXLS (Compact x-ray Light Source) and CXFEL (Compact x-ray Free Electro Laser) sources under development at Arizona State University (ASU). Advanced manufacturing techniques, such as 3D printing of micrometer-sized structures over large areas, are expected to play a critical role for high-throughput sample handling/introduction and automation in the future and, thus, will accelerate discoveries in the biological (and materials) sciences with XFELs and synchrotrons.

## Data Availability

The data that support the findings of this study are available from the corresponding author upon reasonable request.
